# The Effects Induced by Microwave Field upon Tungsten Wires of Different Diameters

**DOI:** 10.3390/ma14041036

**Published:** 2021-02-22

**Authors:** Marian Mogildea, George Mogildea, Valentin Craciun, Sorin I. Zgura

**Affiliations:** 1 Institute of Space Science, MG-36, 077125 Magurele, Romania; marian_mogildea@spacescience.ro (M.M.); szgura@spacescience.ro (S.I.Z.); 2 National Institute for Laser, Plasma and Radiation Physics, Laser Department, 409 Atomistilor St., 077125 Magurele, Romania; valentin.craciun@inflpr.ro; 3 Extreme Light Infrastructure for Nuclear Physics, ELI-NP, HH-IFIN, 077125 Magurele, Romania

**Keywords:** microwave field, plasma, wave guides, metallic wires, electric resistance

## Abstract

The effects induced by microwave field upon tungsten wires of different diameters were investigated. Tungsten wires with 0.5 and 1.0 mm diameters were placed in the focal point of a single-mode cylindrical cavity linked to a microwave generator and exposed to microwave field in ambient air. The experimental results showed that the 0.5 mm diameter wire was completely vaporized due to microwaves strong absorption, while the wire with 1 mm diameter was not ignited. During the interaction between microwaves and tungsten wire with 0.5 mm diameter, a plasma with a high electronic excitation temperature was obtained. The theoretical analysis of the experiment showed that the voltage generated by metallic wires in interaction with microwaves depended on their electric resistance in AC and the power of the microwave field. The physical parameters and dimension of the metallic wire play a crucial role in the ignition process of the plasma by the microwave field. This new and simple method to generate a high-temperature plasma from a metallic wire could have many applications, especially in metal oxides synthesis, metal coatings, or thin film deposition.

## 1. Introduction

The propagation of electromagnetic radiation in different media has been a topic of great interest both for science and industry. It is known that the absorption and reflection processes of the microwaves strongly depend on the material properties. Gases and liquids can absorb the microwaves [1,2,3], while bulk metals reflect them [4]. 

In 2000, Whittaker et al. [5] showed that metal powders mixed in a liquid media and exposed to 600 W microwave these can generate electrical arcing, having as result the heating of the liquid through ohmic effect.

In 2001, Chen et al. [6] showed that metals in powder form can become microwave absorbent. Using the TE_103_ single-mode cavity coupled to a microwave source, metal powders were heated under a nitrogen atmosphere. 

To evaluate microwave absorption efficiency of the metallic powders, Mondal et al. [4] exposed copper powders with different particles size to a microwave field. In their experiment, the powders reached a temperature of 1200 °C for 6 μm particles size, while metal powders with 383 μm particles size reached only 800 °C.

The temperature values of the metallic powders obtained in interaction with microwaves were attributed to the skin depth ($\delta_{S}$) value of the particles. In their work the authors highlighted that metallic particles with 6 μm particle size are better microwave absorbent. Other similar experiments were performed by Popescu S. et al. [7]. Using two metallic pieces (a titanium electrode and a titanium plate) brought into contact when irradiated in a microwave field, the authors managed to generate a plasma in atmospheric air. Therefore, not only metal powders can become microwave absorbent, but under certain conditions, metal wires can also absorb microwaves. 

Since 2010 we have conducted several studies on microwaves interaction with electrically isolated metallic wires. The experimental studies showed that metal wires much thicker than the material skin depth value can absorb the microwaves [8,9,10] having as an effect the generation of a plasma. In our experiments we also observed that not all metallic wires can be ignited by the microwave field. 

Taking into account the current scientific literature, the aim of this work is to investigate the microwave ignition process of the metallic wires as a function of their diameters and the plasma characterization generated in this process.

Therefore, using a commercially available magnetron coupled with a cylindrical cavity we irradiated two types of wires with microwaves: tungsten wire of 5 cm length with 0.5 mm diameter and tungsten wire of 5 cm length with 1.0 mm diameter. 

The experiment was realized in air at normal pressure. The experimental results showed that the tungsten wire with 0.5 mm diameter was vaporized in interaction with the microwave field, while the tungsten wire with 1 mm diameter was not ignited by microwaves. To determine the voltage induced by microwaves in these tungsten wires we calculated the electric voltage as a function of their electric resistance in AC (alternating current) and the power of the microwaves.

The theoretical results showed that the metallic wire with 0.5 mm diameter exposed at 800 W microwave power emitted by magnetron can easily reach the breakdown voltage value in air. Moreover, in microwave discharge process, the tungsten wire with 0.5 mm diameter generated a self-sustaining plasma. Using optical emission spectroscopy method (OES) we found that the metallic wire and gas (air) around of the metallic wire was ionized.

## 2. Materials and Methods

**Figure 1:**
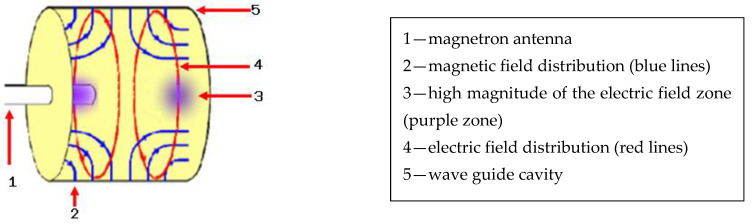
Electromagnetic field propagation in TM_011_ wave guide.

To study the behavior of metallic wires in interaction with the microwaves we exposed them to a high-power microwave field. Using a commercially available magnetron (frequency (*f*) = 2.45 GHz, microwave power = 800 W, (Whicepart Electronic LTD, model 2M2013-01TAG, Zhejiang, China)) coupled with a waveguide cavity we obtained a high power of microwaves in the node zone inside of the waveguide. The metallic wires were placed in this zone and then irradiated with microwaves. The single-mode waveguide was constructed in accordance with TM_011_ propagation mode (transverse magnetic mode) [11], its dimensions being 10.5 cm diameter and 11 cm length; the microwaves entering this waveguide were focused in a single point corresponding to the node of the electromagnetic field (Figure 1). 

The focal point of the wave guide is located in the center of the wave guide at 6 cm (half-wavelength) from the magnetron antenna [12]. The electric field amplitude in this focal point has a very high magnitude, while the dimension of the focal volume is estimated to be around $1{\;\mathrm{cm}}^{3}$. 

The power flux of the electromagnetic field generated by an antenna is defined by the Poynting vector [13,14]:

(1) $$\vec{S}=\frac{1}{2}\;Re\;\left(\vec{E}\;\times \vec{H}\right)\;,\;\left(W/m^{2}\right)$$

According to Figure 1 [15] the 800 W microwave power emitted by the magnetron antenna will be focused in the focal point of the waveguide, thus obtaining around $8\;\mathrm{MW}/{\mathrm{cm}}^{2}$ in this zone. Since the wave impedance of vacuum or dry air is $Z_{w}=376.7\;\Omega$, the electric field (*E*) in this zone can reach a value of:

(2) $$E=\sqrt{2\times S\times Z_{w}}\;=\;7.7\;\mathrm{MV}/m\;\mathrm{or}\;77\;\mathrm{kV}/\mathrm{cm}$$

**Figure 2:**
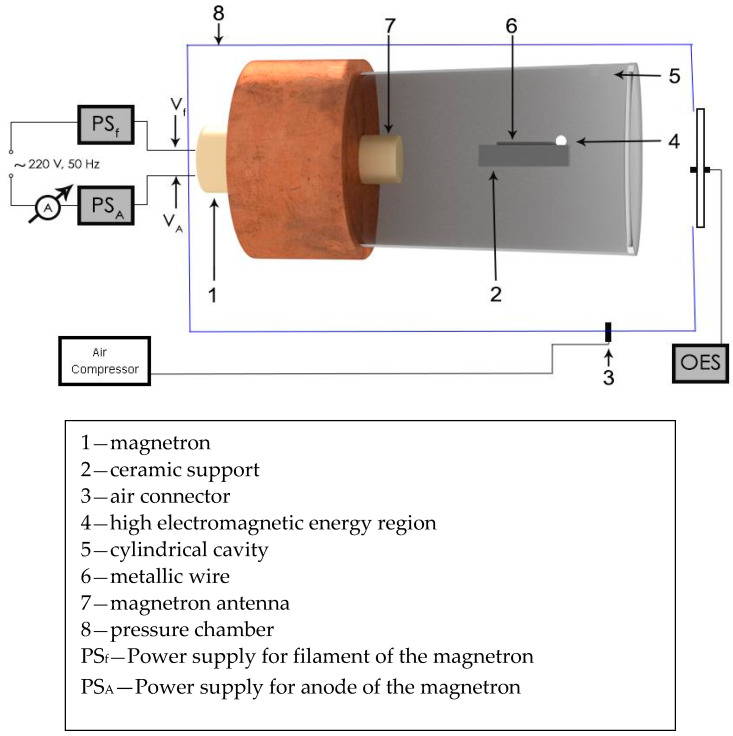
Design of the microwave generator [17].

If a noncontact metallic wire is introduced in the focal point of the single-mode wave guide (Figure 2), the microwave field will induce a strong electric field. If the wire is in air or other gas atmosphere the high electric field can rip an electron of an air molecule creating an ionized molecule and a high energy electron, which could through collisions to ionize other molecules. Ionized atoms strongly absorb microwaves, so once a spark appears, the absorption of the microwave power by the ionized medium will rapidly grow causing the generation of a plasma. In the ionization process of gases from electric discharge or microwave discharge, free electrons are generated. According to the Townsend discharge process, the electrons face collisions with gas molecules and the metallic wire causing vaporization of the wire, excitation, dissociation, electron attachment, or ionization of the gas molecules and the metallic vapors [16]. 

Given that the distance between successive nodes of the TM cylindrical cavity is 6 cm for *f* = 2.45 GHz, the length of the tungsten wires exposed to microwaves was chosen so that they were smaller than the distance between the magnetron antenna and the first node of the waveguide. Tungsten wires were chosen because they have a good electrical conductivity and are slowly vaporized. Also, being a hard material with a very high melting temperature, the wires will not move away from the focal point during vaporization process. To start the experiment, we placed the metallic wire along the cavity symmetry axis with one end in the focal point of the wave guide as schematically shown in Figure 2. The microwave source was turned on. Under these conditions, the tungsten wires were each exposed for 10 s at 800 W microwave power generated by the magnetron. Following the interaction between the microwave and the tungsten electrode, we analyzed the plasma composition through the OES method [18,19] using an Ocean Optics USB 2000 spectrometer (Ocean Optics Inc., Orlando, FL, USA). The optical emission spectrum of the plasma was recorded at 1 ms integration time. The optical resolution gives by the datasheet of the Ocean Optics USB 2000+ spectrometer is: FWHM~0.1 nm [20]. The spectrometer was calibrated using a broadband light source (Ocean Optics DH-mini UV-Vis-NIR Deuterium-Halogen Light Source) before starting the plasma characterization. 

To identify each chemical element (W, N and O) from plasma and correct the emission spectrum of the plasma, we compared our experimental results to three of the most intense spectral lines for each element [21] from the National Institute of Standards and Technology (NIST) data base [22].

## 3. Results 

Our experiments showed that the metallic wires can ignite following microwave absorption processes. Not all metallic wires can absorb the microwaves and create a plasma during interaction process. 

This conclusion was reached after we exposed to microwaves tungsten wires with different diameters. During the experiment we observed that tungsten wires of only 0.5 mm diameter or less generated plasma, while the 1 mm diameter tungsten wires reflected the microwaves. For the initiation of the plasma in air at atmospheric pressure there must be a high voltage induced in the wire. It is impossible to measure the voltage and current generated in the tungsten wire because the presence of a probe inside the microwave field can influence the result of the measurement. To evaluate the voltage induced by microwaves in metallic wires, we calculated the electric resistance (*R*_AC_) in AC of the tungsten wires using the following parameters: the frequency of the electromagnetic radiation, physical properties of the material (magnetic permeability and electrical conductivity) and the length and diameter of the metallic wire. 

Knowing that the impendence (*Z*) of the metallic wire is [23]:

(3) $$Z=\frac{\tilde{V}}{\tilde{I}}\approx \frac{1+j}{{\sigma \delta }_{s}}\times \frac{l}{2\pi a};\;\delta_{s}\ll a–\mathrm{for}\;a\;\mathrm{good}\;\mathrm{conductor}$$

we determined that

(4) $$R_{\mathrm{AC}\;}:\;R_{\mathrm{AC}}=\frac{l}{\sigma \left(\delta_{s}2\pi a\right)};\;\delta_{s}\ll a–\mathrm{for}\;a\;\mathrm{good}\;\mathrm{conductor}$$

If the current density is significant throughout the wire, including along the axis of the wire, δs can be approximated by $\frac{1}{\sqrt{\pi f\mu \sigma }}$. 

In this case *R*_AC_ is:

(5) $$R_{\mathrm{AC}}≃\frac{1}{2}\sqrt{\frac{\mu f}{\pi \sigma }}\;\times \;\frac{l}{a}$$

for tungsten µ ~ 1, σ = 2 × 10^7^ S/m, *f* = 2.45 GHz [24,25].

Where *Z* is the impedance of the metallic wire, µ is the magnetic permeability, σ is the electric conductivity, *f* is the frequency of the electromagnetic radiation, *l* is the length of the metallic wire, and a is the radius of the metallic wire.

The *R*_AC_ for the two metallic wires are: 624 Ω for 0.5 mm tungsten wire and 312 Ω for 1.0 mm tungsten wire. Because the metallic wire can be associated with a resistor we can determination the voltage generated by the wire using the Ohm law:

(6) $$P=V\times I=\frac{V^{2}}{R}=\;\Rightarrow \;V=\;\sqrt{P\times R\;}$$

where *P* is the microwave power in the node region (8 MW), *R* is the *R*_AC_, and *V* is the electric voltage.

The voltage induced by microwaves in the two tungsten wires:

1. Tungsten wire 5 cm length with 0.5 mm diameter: ~71 kV
2. Tungsten wire 5 cm length with 1 mm diameter: ~49 kV

**Figure 3:**
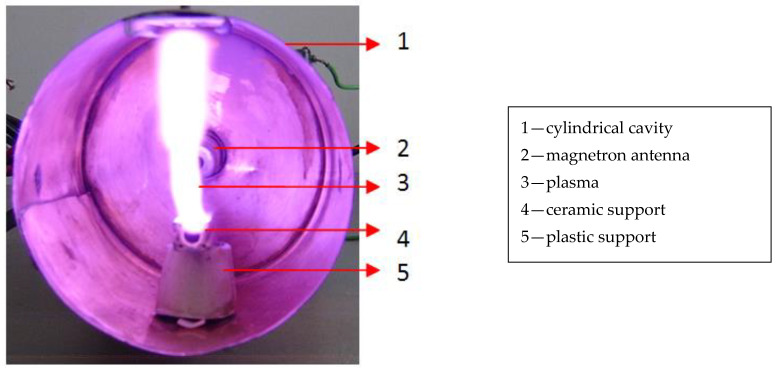
Image of the generated plasma when the 0.5 mm diameter metallic wire was exposed to the microwave field.

In Figure 3, an image of generated plasma due to the interaction of the tungsten wire of 0.5 mm diameter with the microwave field is presented. 

**Figure 4:**
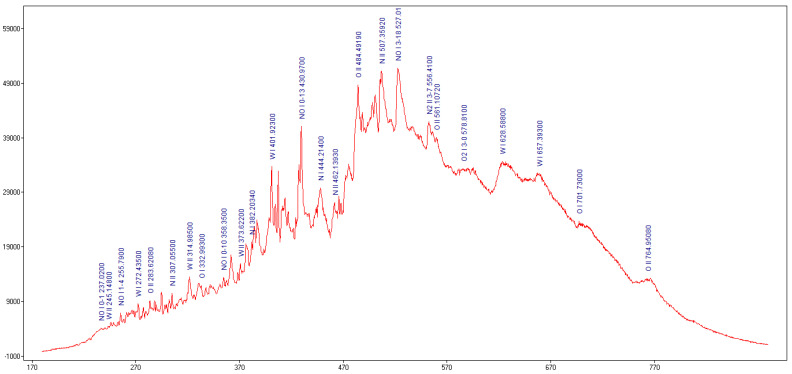
The optical emission spectrum of the generated plasma from a 0.5 mm W wire in air at atmospheric pressure. Wavelength (nm).

To identify the excited neutral atoms species (nitrogen (NI), oxygen (OI), tungsten (WI)) and the ions species (nitrogen (NII), oxygen (OII), tungsten (WII)) [26] from plasma we used the OES method. In Figure 4 the emission spectrum of the plasma at atmospheric pressure recorded from the 0.5 mm W wire plasma is displayed. 

Span V.1.7 spectrum Analyzer software [27] was used to identify the optical emission spectra of the plasma. From optical emission spectrum (Figure 4) we observed that in microwave discharge the WI, WII, NI, NII, OI and OII species were generated. To evaluate the plasma parameters, we determined the electronic excitation temperature. The electronic excitation temperature of tungsten, nitrogen, and oxygen atoms and ions from the plasma were determined using the Boltzmann plot method, which assumes that local thermodynamic equilibrium (LTE) is met within the plasma [28]. 

To build the Boltzmann plots we compared our experimental results with NIST database and we selected only non-overlapping spectral lines. The energetic domain presented on X axis from the Boltzmann Plots corresponded with the frequency of the spectral lines selected for determining the electronic excitation temperature of the excited neutral atoms and ions species.

## 4. Discussion

The interaction of nonionizing electromagnetic radiation with matter is a subject of interest both for science and technology. It is known that bulk metal reflects microwaves while metal powders are heated through the microwave absorption process. However, our experimental results are based on the ignition of metallic wires and generation of the plasma following the microwave absorption process.

The theoretical results explained the experimental ones that only the metallic wire with a diameter of 0.5 mm can “capture” enough energy from the microwave field to create a breakdown in the atmospheric air. An exact value of the breakdown voltage of the atmospheric air is difficult to estimate because its value depends on many parameters (AC or DC voltage, value if the electric voltage, humidity, pressure, temperature) [29], but our simple estimate was quite close to the predicted value. 

If the microwave power is smaller than 800 W or the wire length is smaller than 5 cm, the metallic wire is very hard to ignite. Once the metallic wire was ignited in microwave discharge process, a self-sustaining plasma was generated. Given that in a microwave discharge process a lot of heat is generated, using a simple physical model we can deduce the conditions of the self-sustaining plasma. The heat generated in microwave discharge leads to an increase of the *R*_AC_ of the metallic wire, having as an effect the increase of the electrical voltage in the focal point of the single mode waveguide [30,31] and then the generation of the self-sustaining plasma. 

Knowing that the plasmas are created between an anode and a cathode, in our experiment the noncontact tungsten wire can be considered to be the anode and the walls of the cylindrical cavity the cathode. The plasma was created in air between one end of the tungsten wire from the focal point of the wave guide and the wall of the wave guide, as can be seen in Figure 3. The distance between noncontact metallic wire and wave guide cavity was 5.25 cm. 

If we refer to this distance, the theoretical value of the electric voltage generated by the 0.5 mm metal wire is around ~14 kV/cm. Given that the breaking voltage in air is 20–30 kV/cm for DC voltage [32] according to the Paschen’s law [33] and for AC the breaking voltage decreases with an increase of the frequency [34], our theoretical results are quite close to previous experimental estimations.

The optical emission spectrum (Figure 4) obtained in interaction of the microwaves with metallic wire is similar to the profile of the optical emission spectra obtained in high voltage electric discharges [35]. From the Boltzmann Plot results we noticed that in microwave discharge we obtained a thermal plasma for the excited neutral atoms and ions species. The electronic excitation temperatures of tungsten, nitrogen and oxygen species are shown in Table 1.

**Figure 5:**
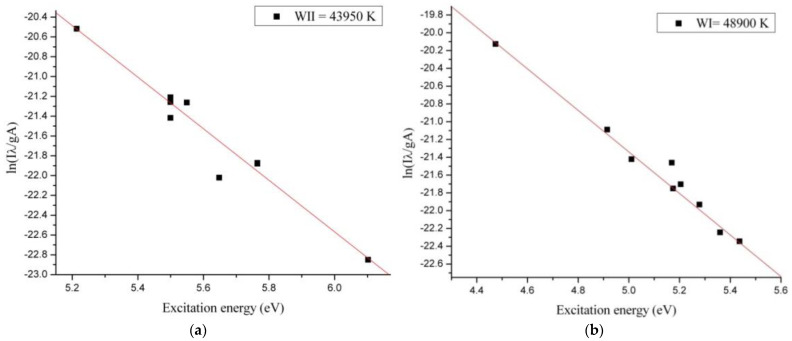
Boltzmann Plot for (**a**) WI in the air at normal pressure and (**b**) for WII in air at normal pressure.

**Figure 6:**
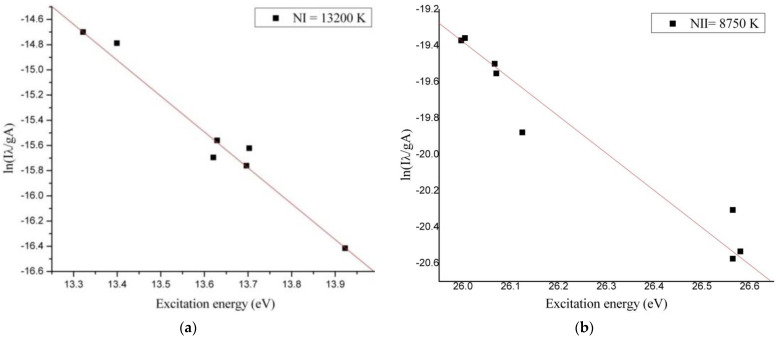
Boltzmann Plot for (**a**) NI in the air at normal pressure and (**b**) for NII in air at normal pressure.

**Figure 7:**
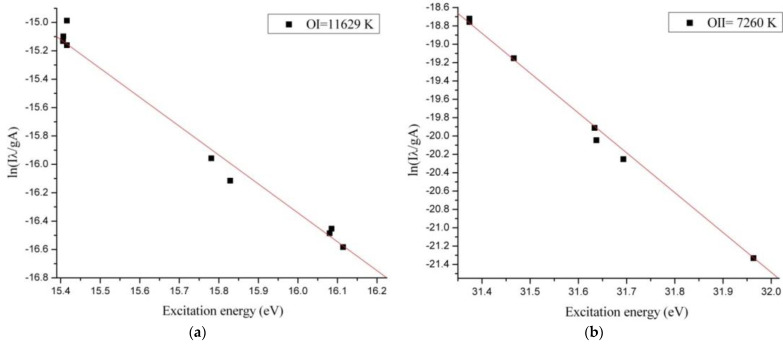
The Boltzmann Plot for (**a**) OI in the air at normal pressure and (**b**) for OII in air at normal pressure. I: intensity of spectral lines, λ: wavelength of spectral lines, A: transition probability, g: the statistical weight of upper energy level, Excitation energy (eV): energy level of the upper state.

Since the electronic excitation temperature of an element from plasma depends on the excitation energy of the element, in our microwave discharge the electronic excitation temperatures of the WI and WII species were much higher than electronic excitation temperature of the OI, OII and NI, NII species (Figure 5, Figure 6 and Figure 7). If we compare the electronic excitation temperatures of the excited neutral atoms with electronic excitation temperatures of the ions, we observe that the electronic excitation temperature of the excited neutral atoms is greater than electronic excitation temperature of the ions. 

Knowing that the electronic excitation temperature value depends by relation $\ln\left(\frac{I\lambda }{Ag}\right)/E\left(\mathrm{eV}\right)\;$ [36] and the ionization energy is always greater than excitation energy, the electronic excitation temperatures of the ion species are smaller.

The ignition of the tungsten wire with 0.5 mm diameter at 800 W microwave power is a repeatable phenomenon and the emission spectrum does not change over time.

In a simple visual analysis between the distribution of the electric field from TM_011_ waveguide (Figure 1) and generation of the plasma at atmospheric pressure (see Figure 3), we observed that the plasma did not have the same shape as the distribution of the electric field. The metallic wire does not disturb the mode of the propagation of the electric field from waveguide. However, experimental results performed in vacuum condition showed that the plasma shape was very close to the shape of the distribution of the electric field from TM_011_ waveguide [9]. Therefore, at atmospheric pressure the circulation of air heated from waveguide during microwave discharge process changes the shape of the plasma. 

In brief, the results of this work showed that the noncontact metallic wires can become microwave absorbent, and the ignition voltage of metal wires depends on the power of the microwaves and the $R_{\mathrm{AC}}$ value of the metallic wire.

## 5. Conclusions

Using a commercial magnetron coupled with a single mode wave guide, we were able to generate a high temperature plasma from a W wire placed in the focal point of the waveguide. The microwave power, dimension and physical properties of the metallic wire play a crucial role in the ignition process of the plasma by the electromagnetic field. A metallic wire with 5 cm length can ignite a plasma at 800 W microwave power generated by the magnetron only if the value of the $R_{\mathrm{AC}}$ of the wire is ≥624 Ω. 

Once the plasma was ignited, very high temperatures were recorded for excited tungsten atoms and ions in air. The plasma is self-sustained because the heat generation in microwave discharge process leads to an increase of the $R_{\mathrm{AC}}$ of the metallic wire having as effect the increase of the electrical voltage from focal point of the single mode wave. 

## Figures and Tables

**Table 1:** The electronic excitation temperature of the excited neutral atoms and ions species.

WI	WII	NI	NII	OI	OII
48,900 K	43,950 K	13,200 K	8750 K	11,629 K	7260 K

## Data Availability

The data presented in this study are openly available.
